# Evaluation of Wound Closure Outcomes Using Barbed Delayed Absorbable Polydioxanone Sutures After Emergency Laparotomy: An Observational Study

**DOI:** 10.7759/cureus.77257

**Published:** 2025-01-10

**Authors:** Aditya Tolat, Arya S V, Raj Kumar Chejara, Dheer S Kalwaniya, Anant Singh, Vakulabharanam Naga Rohith, Pawan Gurivelli, Reena Meena

**Affiliations:** 1 General Surgery, Vardhman Mahavir Medical College and Safdarjung Hospital, New Delhi, IND

**Keywords:** abdominal wound dehiscence, barbed, complication, incisional ventral hernia, laparotomy, polydioxanone, rectus sheath, surgical site infection(ssi), suture, burst abdomen

## Abstract

Background and objectives: There is much debate regarding the ideal suture material for the closure of emergency midline laparotomy wounds. The novel barbed delayed absorbable suture is designed to overcome the shortcomings of conventional suture materials. We conducted a study in 140 patients undergoing emergency laparotomy where barbed delayed absorbable suture was used for rectus sheath closure and observed the time taken for closure and the incidence of postoperative complications like surgical site infection, wound dehiscence, suture sinus formation, and incisional hernia.

Methods: Barbed delayed absorbable (polydioxanone) suture was used for rectus sheath closure of 140 patients undergoing emergency laparotomy using a 4:1 ratio of suture to wound length. Postoperative complications were observed.

Results: Surgical site infection occurred in 40% (56 out of 140) of patients. By postoperative day 8, 70.7% (99 out of 140) of patients had a healthy suture line, 23.6% (33 out of 140) required skin sutures to be opened, and 5.7% (8 out of 140) had burst abdomen. One patient (0.7%) developed suture sinus formation, and five patients (3.6%) were diagnosed with incisional hernia at six months.

Interpretation and conclusion: Barbed delayed absorbable suture can be used for emergency laparotomy wound closure with satisfactory outcomes. It allows faster wound closure without the need for an assistant.

## Introduction

Laparotomy using the midline approach is the most common technique for abdominal incision because it is quick and simple, allows access to all four quadrants, and involves minimal blood loss. The method of abdominal closure after surgery has consistently been a topic of debate. An ideal closure should be simple and durable and act as a barrier to infection. To prevent ischaemia, it should be tension-free and comfortable for the patient [1]. Wound complications following laparotomies are a major cause of postoperative morbidity. Examples include wound infections, incisional hernias, and burst abdomen (wound dehiscence).

Although non-absorbable sutures such as nylon and polypropylene were previously the preferred choice for rectus sheath closure after laparotomy, the most commonly used closure technique for midline laparotomy today is mass closure with delayed absorbable sutures (polydioxanone (PDS)). Absorbable materials are designed to approximate the sheath during the crucial early healing phase and are absorbed later to avoid issues such as sinus formation, discomfort, and buttonhole hernias associated with non-absorbable sutures. PDS sutures lose 50% of their mechanical strength in about three weeks and are fully degraded after approximately six months [2].

The debate over the optimal technique for abdominal fascia closure continues, with most surgeons relying on their own experience rather than evidence-based practice. This approach has resulted in an unchanged frequency of incisional hernias over the past decades [3].

Jenkins' "spring coil effect theory" revolutionised the concept of abdominal wound closure following a midline laparotomy. This theory focuses on aponeurosis closure using continuous bites (without locking) that are more than 1 cm wide and 1 cm apart ("1×1 cm without locking and tension"). This creates a spring coil that accommodates wound distension during the immediate postoperative period. The spring coil facilitates healing using type I collagen, which is found approximately 1 cm from the edge of the rectus sheath [4].

Surgical knots are the weakest part of the suture and reduce its tensile strength. Knots may slip or become overly tightened, leading to localised tissue hypoxia. A recently popularised suture material, the knotless barbed suture, has directional barbs that secure the suture and prevent slippage once it is placed in the tissue. Knotless barbed sutures may reduce operating time and eliminate the need for knots, thereby avoiding their inherent weaknesses [5]. The advantages of this design for tissue approximation include no need for an assistant’s hand to follow suture placement [6]. By eliminating the bulky knots required in conventional wound closure techniques, barbed sutures may reduce the rate of suture extrusion [7].

A review article was published in 2017 describing the design and performance characteristics of the STRATAFIX™ SYMMETRIC PDS™ Plus Knotless Tissue Control Device (Ethicon, Inc., Cincinnati, Ohio) [8]. Their findings were as follows.

Tensile strength

The size 1 STRATAFIX SYMMETRIC PDS Plus Devices had a maximum average tensile strength of 19.16 ± 0.6 lb, while the PDS™ Plus suture had a maximum of 14.28 ± 0.7 lb.

Fixing the suture

In order to secure the proximal and distal ends of a continuous suture pattern and each stitch of an interrupted suture pattern, the conventional suturing technique calls for surgical knots. Knots are a source of additional foreign body bulk. Compared to conventional knots, the STRATAFIX SYMMETRIC PDS Plus Device's fixation tab, which fastens the closure's proximal end, adds much less mass.

Initiation stitch strength

In comparison to the five-throw PDS Plus knot, the STRATAFIX SYMMETRIC PDS Plus Device obtained a statistically greater maximum initiation stitch strength for both sizes 0 and 1.

Wound holding strength

When tested using an INSTRON mechanical testing instrument (Norwood, Massachusetts), the STRATAFIX SYMMETRIC PDS Plus Device demonstrated statistically higher maximum tissue holding forces than both Coated VICRYL Plus Suture (Ethicon, Inc., Cincinnati, Ohio) and PDS Plus Looped Suture [8].

Eighteen patients had abdominal wall repair with STRATAFIX (CTB-1, taper point, suture size 1) in a different trial carried out in 2017. Ten female patients had deep inferior epigastric artery perforator (DIEP) flaps, while eight patients (seven male and one female) had vertical rectus abdominis myocutaneous (VRAM) flaps. "The tissue is far less prone to rip," they concluded. Furthermore, no help is required to seal wounds with high tension since there is very little backsliding of the suture thread, negating the necessity for pulling [9].

A prospective, randomised clinical trial was performed in 2018 in patients undergoing emergent surgery. Aponeurotic closure with triclosan-coated barbed suture (Stratafix Symmetric, Johnson & Johnson), closure with triclosan-coated polydioxanone loop suture (PDS plus [Johnson & Johnson]), and closure with polydioxanone loop suture (PDS) were the three groups into which patients were randomly assigned. It was determined that the incidence of incisional SSIs is decreased when triclosan-coated sutures (Stratafix Symmetric and PDS plus) are used in urgent surgery. The incidence of evisceration is decreased by the use of barbed sutures [10].

A study was conducted in 2015 on 1011 patients to determine whether the incidence of complications differed after wound approximation in plastic surgery when various barbed vs non-barbed sutures were employed. The results showed that the two-layer technique with barbed sutures was associated with significantly higher rates of wound separation than traditional methods [11].

As there is a paucity of Indian studies regarding the efficacy of barbed delayed absorbable sutures for abdominal wound closure after laparotomy, we undertook this study.

## Materials and methods

Aim

The study aims to observe the incidence of laparotomy-related wound complications using barbed delayed absorbable (polydioxanone) sutures for abdominal wall closure after laparotomy.

Primary objective

The primary objective is to evaluate barbed polydioxanone suture for abdominal wound closure after laparotomy with respect to rectus sheath closure time, incidence and grade of surgical site infection according to the Southampton Wound Grading System [12], and incidence and grade of wound dehiscence according to the World Union of Wound Healing Societies (WUWHS) Surgical Wound Dehiscence (SWD) Sandy Grading System [13].

Secondary objective

The secondary objective is to observe the incidence of suture sinus formation and the incidence of early incisional hernia up to a period of six months in the postoperative period.

Study design

This prospective observational study was conducted in the Department of Surgery, Vardhman Mahavir Medical College and Safdarjung Hospital, New Delhi, from December 2020 to May 2022.

Ethical considerations

Ethical clearance was obtained from the institutional ethics committee prior to the commencement of the study. Written informed consent was taken from each participant with the option to drop out of the study at any time.

Inclusion and exclusion criteria

The study included patients aged more than 12 years undergoing emergency laparotomies with midline vertical incisions and having an Acute Physiology and Chronic Health Evaluation II (APACHE II) score [14] of less than 10 in the Department of General Surgery, Safdarjung Hospital, New Delhi, during the study period. The study excluded patients who had a history of previous abdominal surgery, were pregnant, had frank purulent peritonitis or any perforation beyond 48 hours, had raised intra-abdominal pressure, or had malignancy.

Method

After taking a detailed history and completing the examination, all relevant investigations were conducted to confirm the diagnosis. Data on the individuals' co-morbidities, scheduled operation type, and disease type were gathered after obtaining their consent to participate in the study. Once the patient consented to the procedure, they were moved to the operating room following optimal resuscitation.

Surgical technique

Using a midline vertical incision, a laparotomy was performed, and the main pathology was corrected. Depending on the level of contamination during surgery, the laparotomy wounds were classified using the Centers for Disease Control and Prevention (CDC) wound classification [15]. Following each laparotomy, the patients' abdomens were closed using the continuous mass closure approach, which involved full-thickness bites of the linea alba fascia, including the anterior and posterior rectus sheath, with a 4:1 ratio of suture length to wound length. Bites were taken 1 cm from the wound edge and 1 cm away from the previous bite.

In all patients, barbed delayed absorbable polydioxanone suture (No. 1 Stratafix PDS Plus suture) was used. The time required for rectus sheath closure was noted, and the length of the wound was measured after closure. Depending on the level of contamination, the skin was either left open or sutured with No. 2-0 nylon or skin clips. Sterile dressings were applied after closure. The laparotomy suture line was checked after 48 hours and assessed for any early wound complications. Thereafter, the wound was examined daily for surgical site infection (using European CDC criteria [16]) and graded using the Southampton grading system [12]. Wound dehiscence was graded using the WUWHS SWD grading system [13], and suture sinus formation was observed. The wound was re-examined after three months and six months for any late complications, such as incisional hernia. Stitch sinus was defined as a blind-ending tract created by antigenicity or an infective process around a retained suture, leading from the stitch to the skin surface, where serous or purulent material may discharge [17]. An incisional hernia was defined as one observed clinically or radiologically at the location of the midline vertical laparotomy incision [1].

Sample size calculation

The study included 140 patients. Previously, researchers [10] conducted a study to determine the incidence of wound-related complications using barbed delayed absorbable sutures in patients after laparotomy. The complication rate in this study ranged from 5% to 15%. Therefore, assuming (p) = 10% with a 5% margin of error, the minimum required sample size at a 5% level of significance was calculated to be 138 patients.

Statistics

Descriptive analysis was carried out by frequency and proportion for categorical variables. For continuous variables, descriptive statistics were presented as mean (± standard deviation). The chi-square test was used to test the statistical significance of cross-tabulation between categorical variables. Since there was zero or low frequency in some categories in most of the contingency tables, a simulated p-value was calculated wherever required.

## Results

A total of 140 patients were included in the study. The mean age of the study population was 34.13 years. Among the 140 patients, 82.9% (n = 116) were men and 17.1% (n = 24) were women. The majority (24.3%) of the patients were diagnosed with ileal perforation, followed by intestinal obstruction (17.9%) and pre-pyloric perforation (12.1%). Most (57.1%) of the wounds were classified as dirty, 28.6% were classified as clean, 7.9% were classified as clean-contaminated, and 6.4% were classified as contaminated. The mean (± SD) wound length in the study population was 17.44 (± 1.54) cm. The mean (± SD) time for rectus sheath closure was 10.30 (± 1.40) minutes. There was a statistically significant difference in the proportion of time for rectus sheath closure according to wound length, with longer wounds requiring more time for closure. The mean (± SD) duration of surgery was 2.26 (± 0.54) hours. Postoperatively, 40% of the patients developed some form of surgical site infection. By postoperative day 8, 70.7% of the patients had a healthy suture line, 23.6% had the suture line skin (and subcutaneous tissue) opened, and 5.7% experienced a burst abdomen (Table 1).

**Table 1:** POD 8 suture line status in the study population (N=140) POD: postoperative day.

POD 8 suture line status	Frequency (percentage)	
Healthy	99 (70.7%)	
Open	33 (23.6%)	
Burst	8 (5.7%)	

There was a statistically significant difference in the proportion of Southampton grades according to wound classification (p < 0.05). Among patients with clean wounds, 87.5% had normal healing, compared to 47.5% among dirty wounds, 72.7% among clean-contaminated wounds, and 33.3% among contaminated wounds. There was a statistically insignificant difference in the proportion of Southampton grades according to age group, gender, and duration of surgery. There was a statistically significant difference in the proportion of WUWHS grades according to wound classification (p < 0.05). Among patients with clean wounds, 95% had no dehiscence, compared to 57.5% among dirty wounds, 81.8% among clean-contaminated wounds, and 66.7% among contaminated wounds. There was a statistically insignificant difference in the proportion of WUWHS grades according to age group, gender, and duration of surgery (p > 0.05). However, among the 140 patients, only one patient (0.7%) developed a suture sinus (Table 2).

**Table 2:** Among 140 patients, only 1 patient (0.7%) had suture sinus

Suture sinus	Frequency (percentage)	
Yes	1 (0.7%)	
No	139 (99.3%)	

 Five patients (3.6%) had incisional hernia at six months (Table 3).

**Table 3:** Incisonal hernia in the study population (N=140)

Incisonal hernia	Frequency (percentage)	
Yes	5 (3.6%)	
No	135 (96.4%)	

All five cases developed a burst abdomen postoperatively. However, there was a statistically insignificant difference in the proportion of incisional hernia according to age group, wound classification, gender, and duration of surgery.

## Discussion

We observed that the mean age of the patients undergoing emergency laparotomy was 34.13 years. Similar results were reported by Gangwal et al. [18] (mean age of 36.1 years) and Singal R et al. [2] (mean age of 36.7 years). This finding is worrisome as laparotomy and its complications are likely to hamper the productivity of this young age group in India.

In our study, laparotomy was more common in men. This male preponderance is comparable to other studies by Singal et al. [2] (male: female = 2.16), Pai et al. [1] (62% males, 32% females), and Kailas et al. [19] (male: female = 1.94). The male preponderance could be attributed to increased use of intoxicants such as alcohol, smoking, irregular meals, increased outdoor activity, and consumption of spicy foods, all of which are known to play a role in bowel pathologies. Additionally, polytrauma is more common in men compared to women [19].

The high incidence of ileal perforation in our study can be attributed to the higher prevalence of typhoid and abdominal tuberculosis in India. Similar observations were made by Khanna et al. [20].

Most of the previous studies did not emphasise the classification of wounds. However, the clinical significance of proper wound classification lies in its ability to help predict the likelihood of surgical site infections, postoperative complications, and the need for re-operation [21].

The length of the laparotomy wound is variable, depending on the underlying pathology, which may require a longer or shorter wound for adequate exposure. Wound length is also influenced by the patient’s build. Nonetheless, a longer wound will require more time for closure. According to the suture technique used in our study, an increase in wound length by 1 cm would require an additional bite on each side of the rectus sheath, thereby increasing the time required for closure.

In our study, the mean time for rectus sheath closure was 10 minutes 30 seconds. In a study by Peponis et al. [22], the median time required for rectus sheath closure after laparotomy using polydioxanone No. 1 suture was 13 minutes. The shorter time required for rectus sheath closure when using barbed suture may be attributed to the fixation tab at the end of the suture, which eliminates the need for taking 6-8 throws of square knots that are normally required to fix the suture to one end of the wound when performing the continuous mass closure technique with conventional polydioxanone suture. The knot subsequently needs to be buried, which adds to the time required for wound closure. Another factor contributing to the reduced time for rectus sheath closure is the ability of the barbs to hold the suture in place. This prevents back slippage of the suture and allows the surgeon to focus on the next bite without worrying about loosening the previous one.

We observed that in our study, the mean duration of surgery was 2.26 hours. This is significant because the likelihood of complications increases markedly with prolonged operative duration, approximately doubling when operative time exceeds thresholds of two or more hours [23].

In our study, for any wound exhibiting serous or purulent discharge, the skin sutures were opened to allow adequate drainage and irrigation. Wounds presenting with erythema and contusions were managed with appropriate antibiotics without opening the skin sutures. A total of 70.7% of suture lines remained healthy and intact, 23.6% had the skin sutures opened, and 5.7% experienced a burst abdomen.

All cases of burst abdomen in our study occurred in wounds classified as dirty at surgery. The suture lines in these wounds progressed to develop purulent discharge, which subsequently led to a burst abdomen. This finding suggests an infective cause rather than a technical failure. Infection contributes to aponeurotic breakdown and failure in more than half of all cases of wound dehiscence [24].

The study by Ruiz-Tovar et al. [10] reported the incidence of evisceration as 0% in the Stratafix Symmetric group, 8.9% in the PDS Plus Loop group, and 12.8% in the PDS Loop group. The study by Pai et al. [1] reported the incidence of burst abdomen as 3.6% in the PDS group and 2.6% in the Prolene group. Similarly, the study by Kailas et al. [25] reported an incidence of burst abdomen with no cases in the PDS group and one case (4% incidence) in the Prolene group. This difference may be attributed to the inclusion of both emergency and elective laparotomies in these studies.

These findings indicate that the type of suture material may not have a substantial impact on the incidence of burst abdomen. However, the level of contamination at surgery appears to have a stronger association with burst abdomen.

The low incidence of suture sinus in our study may be attributed to the delayed absorbable nature of the suture material and the elimination of the need to tie multiple fixing knots at the start of rectus sheath closure. These knots, if positioned close to the skin surface, could lead to a suture sinus.

In our study, incisional hernias were observed exclusively in patients who developed burst abdomen and had dirty wound classifications. However, the data were insufficient to establish any statistical association between incisional hernia and variables such as age, sex, duration of surgery, or wound classification. Additionally, due to the time-bound nature of our study, the incidence of incisional hernia may have been under-reported in cases where hernias developed more than six months post-surgery.

## Conclusions

In conclusion, barbed delayed absorbable suture can be used for abdominal wound closure after laparotomy with satisfactory wound-related outcomes. However, our study was a single-centre study with a limited number of participants due to the time-bound nature of the study and specific inclusion and exclusion criteria. A multi-centre, comparative study comparing polydioxanone, barbed polydioxanone, and Prolene is required to determine the ideal suture for abdominal wound closure after laparotomy.

## References

[1] Pai D, Shenoy R, Chethan K (2018). Comparison of non-absorbable (polypropylene) versus delayed absorbable (polydioxanone) suture material for abdominal wound closure after laparotomy. Int Surg J.

[2] Singal R, Kumar M, Kaushik N, Dhar S, Singh B (2016). A comparative study of polydioxanone and nylon for abdominal wall closure with interrupted figure of eight in peritonitis cases. J Curr Surg.

[3] Höer J, Stumpf M, Rosch R, Klinge U, Schumpelick V (2002). Prevention of incisional hernia (Article in German). Chirurg.

[4] Chintamani Chintamani (2018). Editorial: ten Commandments of safe and optimum abdominal wall closure. Indian J Surg.

[5] Peleg D, Ahmad RS, Warsof SL, Marcus-Braun N, Sciaky-Tamir Y, Ben Shachar I (2018). A randomized clinical trial of knotless barbed suture vs conventional suture for closure of the uterine incision at cesarean delivery. Am J Obstet Gynecol.

[6] Paul MD (2009). Bidirectional barbed sutures for wound closure: evolution and applications. J Am Col Certif Wound Spec.

[7] Murtha AP, Kaplan AL, Paglia MJ, Mills BB, Feldstein ML, Ruff GL (2006). Evaluation of a novel technique for wound closure using a barbed suture. Plast Reconstr Surg.

[8] Nawrocki JG, Nonnenmann H, Mooney M, Sutton N, Schmitz ND (2017). A high-strength, absorbable, antibacterial knotless tissue control device for fascial closure. Curr Obstet Gynecol Rep.

[9] Yasuda S, Tomita K, Kiya K, Hosokawa K (2017). STRATAFIX for abdominal wall repair following abdominal flap harvest. Plast Reconstr Surg Glob Open.

[10] Ruiz-Tovar J, Llavero C, Jimenez-Fuertes M, Duran M, Perez-Lopez M, Garcia-Marin A (2020). Incisional surgical site infection after abdominal fascial closure with triclosan-coated barbed suture vs triclosan-coated polydioxanone loop suture vs polydioxanone loop suture in emergent abdominal surgery: a randomized clinical trial. J Am Coll Surg.

[11] Cortez R, Lazcano E, Miller T (2015). Barbed sutures and wound complications in plastic surgery: an analysis of outcomes. Aesthet Surg J.

[12] Bailey IS, Karran SE, Toyn K, Brough P, Ranaboldo C, Karran SJ (1992). Community surveillance of complications after hernia surgery. BMJ.

[13] (2018). Consensus document: surgical wound dehiscence: improving prevention and outcomes. https://woundsinternational.com/world-union-resources/consensus-document-surgical-wound-dehiscence-improving-prevention-and-outcomes/.

[14] Knaus WA, Draper EA, Wagner DP, Zimmerman JE (1985). APACHE II: a severity of disease classification system. Crit Care Med.

[15] Surgical Site Infection (SSI (2010). Surgical site infection (SSI) event: Center for Disease Control. http://www.cdc.gov/nhsn/PDFs/pscManual/9pscSSIcurrent.pdf.

[16] (2015). Surgical Site Infection Event (SSI). https://www.cdc.gov/nhsn/PDFs/pscManual/9pscSSIcurrent.pdf.

[17] Rabiu AR, Tan LC (2016). A complication to remember: stitch sinus following laparoscopic umbilical hernia repair. J Surg Case Rep.

[18] Gangwal M, Singh B (2018). Incidence of post-operative pulmonary complications following emergency laparotomy in tertiary care centre in Vindhya region of Madhya Pradesh, India. Int Surg J.

[19] Singh A, Chejara RK, Sharma AK, Tolat A (2021). Study of the efficacy of trauma and injury severity score to predict survival in patients of polytrauma. Int Surg J.

[20] Khanna AK, Misra MK (1984). Typhoid perforation of the gut. Postgrad Med J.

[21] Mioton LM, Jordan SW, Hanwright PJ, Bilimoria KY, Kim JY (2013). The relationship between preoperative wound classification and postoperative infection: a multi-institutional analysis of 15,289 patients. Arch Plast Surg.

[22] Peponis T, Bohnen JD, Muse S (2018). Interrupted versus continuous fascial closure in patients undergoing emergent laparotomy: a randomized controlled trial. J Trauma Acute Care Surg.

[23] Cheng H, Clymer JW, Po-Han Chen B, Sadeghirad B, Ferko NC, Cameron CG, Hinoul P (2018). Prolonged operative duration is associated with complications: a systematic review and meta-analysis. J Surg Res.

[24] Townsend CM, Beauchamp RD, Evers BM, Mattox KL (2022). Sabiston Textbook of Surgery: The Biological Basis of Modern Surgical Practice. https://shop.elsevier.com/books/sabiston-textbook-of-surgery/townsend/978-0-323-64062-6.

[25] Kailas CT, Naik CG (2017). A randomised control trial comparison the efficacy between delayed-absorbable polydioxanone and nonabsorbable suture material in abdominal wound closure. Int Surg J.

